# A Mini-Review on the Co-growth and Interactions Among Microorganisms (Fungi and Bacteria) From Rhizosphere of Metal-Hyperaccumulators

**DOI:** 10.3389/ffunb.2021.787381

**Published:** 2021-11-24

**Authors:** Grazia Cecchi, Simone Di Piazza, Stefano Rosatto, Mauro Giorgio Mariotti, Enrica Roccotiello, Mirca Zotti

**Affiliations:** ^1^Laboratory of Mycology, Department of Earth, Environment and Life Sciences, University of Genoa, Genoa, Italy; ^2^Laboratory of Plant Biology, Department of Earth, Environment and Life Sciences, University of Genoa, Genoa, Italy

**Keywords:** fungal-bacterial consortia, metallophytes, PTMs removal, rhizobiota, rhizosphere interactions

## Abstract

The co-growth and synergistic interactions among fungi and bacteria from the rhizosphere of plants able to hyper accumulate potentially toxic metals (PTMs) are largely unexplored. Fungi and bacteria contribute in an essential way to soil biogeochemical cycles mediating the nutrition, growth development, and health of associated plants at the rhizosphere level. Microbial consortia improve the formation of soil aggregates and soil fertility, producing organic acids and siderophores that increase solubility, mobilization, and consequently the accumulation of nutrients and metals from the rhizosphere. These microorganism consortia can both mitigate the soil conditions promoting plant colonization and increase the performance of hyperaccumulator plants. Indeed, microfungi and bacteria from metalliferous soils or contaminated matrices are commonly metal-tolerant and can play a key role for plants in the phytoextraction or phytostabilization of metals. However, few works deepen the effects of the inoculation of microfungal and bacterial consortia in the rhizosphere of metallophytes and their synergistic activity. This mini-review aimed to collect and report the data regarding the role of microbial consortia and their potentialities known to date. Moreover, our new data had shown an active fungal-bacteria consortium in the rhizosphere of the hyperaccumulator plant *Alyssoides utriculata*.

## Introduction

Among the non-renewable life-supporting resources, the soil is the main reservoir of potentially toxic metals (PTMs). Many technologies, traditional (chemical-physical-electrical) and not (biological), were developed to decrease the PTMs contamination in soils (Liu et al., 2018). Traditional technologies often have a very negative impact on ecosystems and biodiversity due, for example, to the employment of chemicals or soil excavation (Ali et al., 2013; Lacalle et al., 2020). On the contrary, biological methods are cheap, sustainable, but time-consuming. For this reason, the real application of these methods to date is limited. To cope with this problem, recently, many studies were carried out to improve the performance of biological methods, integrating different biotechnologies (Asad et al., 2019; Alves et al., 2021; Tiodar et al., 2021). For example, phytostrategies benefit from the use of microorganisms, particularly plant growth-promoting bacteria (PGPB) and fungi (PGPF), to remediate contaminated sites (Alves et al., 2021; Tiodar et al., 2021). Microorganisms intervene in biogeochemical cycles, minerals dissolution and/or bioprecipitation, and metals mobilization, and/or immobilizations thanks to their metabolic pathways, organic acids, and enzymes (Choudhary, 2012; Singh and Shourie, 2021). These bio-products can alter the micro-environment in which these microbes live and promote redox and other chemical reactions, also modifying the pH of the substrates (Singh and Shourie, 2021). Hence many studies were developed to investigate the role played by rhizobiota in plant growth promotion and protection (Hao et al., 2021; Khalid et al., 2021). Most plants are not able to mitigate the impacts of environmental stress, depending on microorganisms (mainly fungi and bacteria) (Singh and Shourie, 2021). Beneficial fungal symbioses (mycorrhization and endophytism) not only can allow plants specific habitats colonization, but also the adaptation to many stresses due to the global climatic change (e.g., increase of carbon dioxide [CO_2_], UV radiation, and desertification, among others) (Rodriguez and Redman, 2008; Rodriguez et al., 2008; Choudhary, 2012; Cecchi et al., 2019b; Singh and Shourie, 2021). Together with fungi, bacteria are also known to be plant growth promoters (Basu et al., 2021). In the rhizosphere of metallophytes, these microbes can increase the efficiency of phytoremediation of contaminated ecosystems. PTMs-resistant-PGPB is characterized by the capability to stabilize PTMs reducing their toxicity through physiological and biochemical activities (Harindintwali et al., 2020).

Recently, some studies assessed the role and interactions among bacteria, fungi, and plants in phytomining, and phytoremediation processes (Thijs et al., 2017; Kazemalilou et al., 2020; Alves et al., 2021). However, many works investigate only the group of mycorrhizal fungi, while the role of microfungi associated with plants root is still little studied (Turnau and Mesjasz-Przybylowicz, 2003; Mishra et al., 2016; Alves et al., 2021). Moreover, the interactions between PGPB and non-mycorrhizal fungi are not clear to date. Few studies deepened these interactions and the possible synergism between bacteria and fungi in the rhizosphere of hyperaccumulator plants (Rosatto et al., 2021a).

This mini-review aimed to briefly collect the data regarding the role of bacteria and microfungi in the rhizosphere of PTMs hyperaccumulator plants, reporting data on the interactions between these microorganisms in the rhizosphere and data on how these relations could influence the rate of metals uptake by plants.

## The Role of Bacteria in The Rhizosphere of Metallophytes

To date, the PGP role of rhizosphere bacteria is well-known (Souza et al., 2015; Vejan et al., 2016; Leontidou et al., 2020; Basu et al., 2021). Many *in situ* studies were carried out on various plants species in order to verify the PTMs tolerance and bioaccumulation by PGPB from the rhizosphere (Han et al., 2020; Ali et al., 2021). They can solubilize and mobilize PTMs increasing their availability, altering the soil pH, and inducing redox reactions through the secretion of biosurfactants, organic acids, siderophore, and chelating agents (Rajkumar et al., 2012; Ojuederie and Babalola, 2017). Moreover, PGPB can produce phytohormones [e.g., auxin and indole-3-acetic acid (IAA)] responsible for regulating plants growth and essential in the adaptation to environmental stresses such as PTMs (Rajkumar et al., 2012; Asad et al., 2019; Wagi and Ahmed, 2019). The majority of PGPB were identified as endophytes from PTM-contaminated soils in plant rhizospheres and tissues. The most frequent genera are *Agrobacterium, Bacillus, Brucella, Burkholderia, Escherichia, Mesorhizobium, Pseudomonas, Rhizobium*, and *Streptomyces*. Many authors have shown that coupling bioaugmentation with selected PTM-resistant PGPB and phytoremediation technologies can positively affect the PTMs phytoextraction (Harindintwali et al., 2020; Wang et al., 2021). The role played by native microbes is essential: many reports show that bacterial strains collected from ultramafic soils, associated with Ni hyperaccumulators, tolerate higher concentrations of Ni in comparison to strains from other soils (Turgay et al., 2012; Rosatto et al., 2019). Etesami (2018) evidenced that the inoculation of plants rhizosphere with PTMs resistant strains of PGPB can alleviate plant stress, helping plants to mitigate PTMs toxicity and reducing their accumulation in plant tissues. Kumar et al. (2021), after the isolation of native endophytic bacteria from the tissues of the Ni-hyperaccumulator *Odontarrhena obovata* C.A. Mey., screened the strains about their Cu tolerance and PGP function. The authors not only selected a *Pseudomonas lurida* Behrendt et al. 2007 strain as the most performant in Cu remediation but also tested this strain in the rhizosphere of sunflower. They evidenced that rhizospheric soil added with the bacterium showed an increase in Cu uptake by 8.6-fold for roots and 1.9-fold for leaves than uninoculated plants. Moreover, Khatri et al. (2020) studied Cd tolerance of cold-tolerant and PGP rhizobacteria *Pseudomonas putida* (Trevisan, 1889) Migula 1895 and *Bacillus subtilis*, isolated from the Indian Himalaya Region, and their effects on growth and Cd accumulation in wheat seedlings under mountain ecosystem. They showed that bacteria protect the wheat plants reducing and preventing Cd bioaccumulation in wheat and the food chain, also improving its growth. Regarding Ni hyperaccumulator species, a study from 2003 reported that *Microbacterium arabinogalactanolyticum* (Yokota et al. 1993) Takeuchi and Hatano 1998 has an important role in enhancing Ni availability and therefore its accumulation by *Odontarrhena chalcidica* (Janka) Španiel, Al-Shehbaz, D.A. German, and Marhold (Abou-Shanab et al., 2003; Bani and Echevarria, 2019; Rosenkranz et al., 2019; Dimitrakopoulos et al., 2021; Hipfinger et al., 2021). An increase of 32.4% in shoot Ni concentration was reported after inoculation, in comparison with the uninoculated specimens. Another study, with the same plant species, but with another bacterial strain inoculated, *Microbacterium oxydans* (Chatelain and Second 1966) Schumann et al. (1999), also showed an increase in Ni bioaccumulation (Abou-Shanab et al., 2006). Other studies showed that the inoculation of *Pseudomonas fluorescens* complex strongly increases *Sedum alfredi* Hance biomass, enzyme activities, shoot chlorophyll, and Cd concentration (Chen et al., 2017) as already demonstrated by Chen et al. (2014) and Ali et al. (2017) for IAA-producing endophytic bacteria. Recent studies reveal that *P. fluorescens* significantly enhances the photosynthetic yield in terms of maximum quantum yield of Photosystem II, photochemical quenching, net photosynthetic rates, intercellular CO_2_ concentration, transpiration rate, and stomatal conductance (Wu et al., 2020a). Furthermore, *P. fluorescens* promotes the development of lateral roots and the root-to-shoot transport of Cd, improving the phytoremediation efficiency (Wu et al., 2020b,c).

Among endophytes, *Streptomyces lydicus* De Boer et al. (1956) is known to promote health and plant growth (Worsley et al., 2020), colonizing the root and acting as PGP in pea plant and other legumes (Tokala et al., 2002). However, only certain strains contribute to increasing plant biomass individually and in combination; conversely, other strains appear to inhibit the development of *Arabidopsis thaliana* (L.) Heynh (Worsley et al., 2020).

## The Role of Microfungi in The Rhizosphere of Metallophytes

Most of the studies carried out on the role of fungi in the rhizosphere deal with the mycorrhizal fungi (Turnau and Mesjasz-Przybylowicz, 2003; Mishra et al., 2016; Alves et al., 2021) which supply essential nutrients, increase plant health, and enhance stress tolerance (e.g., drought). Fungi can induce the immobilization of metals on the surface of living hyphae using chemical bonding groups in the cell wall or by complexation of the metals with small molecular organic compounds secreted in the rhizosphere (Thijs et al., 2017; Cecchi et al., 2019a). Concerning hyperaccumulator plants, for example, they were thought to be non-mycorrhizal, therefore, there is still little information in comparison to studies on bacterial inoculants (Khan, 2005; Benizri and Kidd, 2018). The first Ni hyperaccumulator plant in which the presence of arbuscular mycorrhizal fungi was reported was *Berkheya coddii* Roessler (Turnau and Mesjasz-Przybylowicz, 2003; Alves et al., 2021). Moreover, little is still known about the role of microfungi in the rhizosphere of plants able to hyper accumulate PTMs. These associations of the microorganisms can also modify the chemical composition of root exudates and the bioavailability of PTMs in the soil. Zhang et al. (2018) studied the bioremediation mechanisms of Pb and Cd contaminated soil using two indigenous fungi (*Mucor circinelloides* Tiegh. and *Trichoderma asperellum* Samuels, Lieckf. and Nirenberg) selected from mine tailings as the phytostimulation of *A. thaliana* and showed that these microfungi can relieve plant stress improving the phytoremediation activity. Among different *Trichoderma* species, *T. harzianum* Rifai represents the highest metabolic diversity which is associated with its numerous beneficial effects on plants such as growth promotion and enhancement of stress resistance (Zeilinger et al., 2016). Many studies evidenced its role in the rhizosphere of plants able to bioaccumulate and hyper accumulate metals, and also its capability to uptake Ni, Cu, and Ag (Zotti et al., 2014; Roccotiello et al., 2015; Cecchi et al., 2017a,b). Wazny et al. (2021) focused on the role of endophytic fungi in the enhancing of Ni-hyperaccumulation by *Noccaea caerulescens* (J. Presl and C. Presl) F.K. Mey. After the isolation and identification of fungal strains, they inoculated plants with six fungal species. They reported that *Diaporthe columnaris* (D.F. Farr and Castl.) Udayanga and Castl. was able to improve Ni uptake by plants, also activating specific gene networks. Xie et al. (2014) investigated the role of *Aspergillus aculeatus* Iizuka in the rhizosphere of bermudagrass [*Cynodon dactylon* (L.) Pers.] a known species able to bioaccumulate Cd. The inoculation of the plants with this fungal strain showed that *A. aculeatus* improved Cd tolerance and reduced Cd transportation to shoot of bermudagrass. Similarly, Babu et al. (2014) tested *Trichoderma* sp. and *Talaromyces aculeatus* (Raper and Fennell) Samson, N. Yilmaz, Frisvad and Seifert strains, isolated from the rhizosphere of *Pinus koraiensis* Siebold and Zucc. in a mine tailing soil, for their PTMs tolerance and PGP characteristics. They reported that the isolates increased available P in a 1:1 (w/w) mixture of soil and liquid media by 14–43% and the bioavailability of As, Cu, Pb, and Zn, and exhibited phosphatase, phytase, and siderophore activity. In this scenario, Restu and Payangan (2019) studied the production of IAA by some rhizosphere fungal strains. *Fusarium* resulted in the genus with the highest production of IAA, showing how fungi of this genus could be exploited as biological fertilizers. This result is very interesting because *Fusarium* is a well-know parasite/pathogen of crops. However, not all the species belonging to this genus show the presence of the pathogenicity gene (i.e., non-pathogenic strains belonging to *F. oxysporum, F. solani* (Mart.) Sacc. and *F. fujikuroi* Nirenberg species; Al-Ani, 2019), evidencing how the relations between fungi and plants can be very complex and difficult to understand (Zeilinger et al., 2016). Comparative analyses have revealed that the *Fusarium* genome is compartmentalized into regions responsible for primary metabolism, reproduction, pathogen virulence, host specialization, and possibly other functions (Ma et al., 2013). Hence there is the possibility to transfer pathogenic chromosomes to non-pathogenic species (horizontal transfer) within the *Fusarium* genus (Ma et al., 2013).

**Figure 1:**
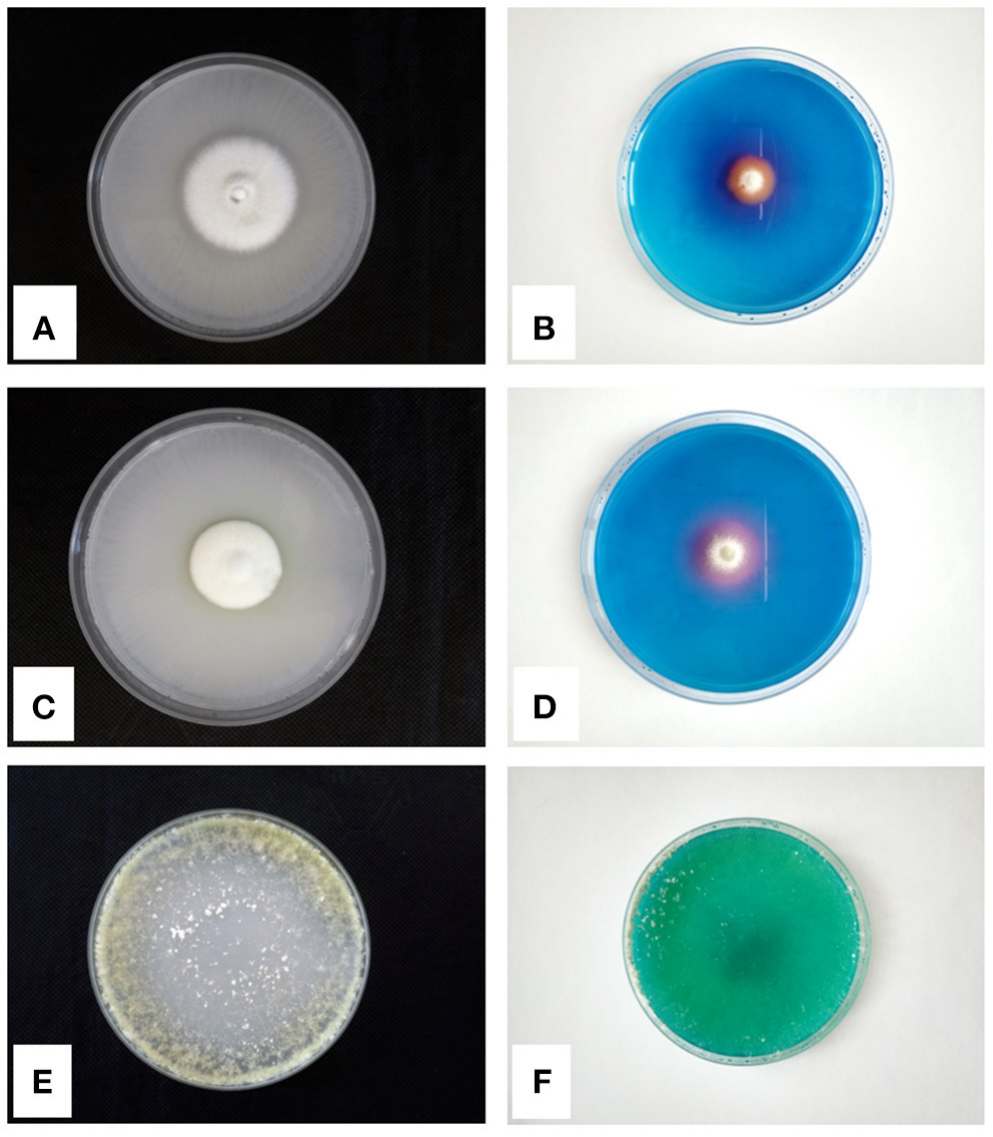
P-solubilization tests on Pikovskaya Agar and siderophores production tests on CAS-Agar of *Penicillium ochrochloron* **(A,B)**, *Penicillium canescens* **(C,D)**, and *Trichoderma harzianum* **(E,F)**, respectively.

Other microfungi, isolated from the rhizosphere of the facultative hyperaccumulator *A. utriculata*, such as *Penicillium canescens* Sopp, *P. ochrochloron*, and *T. harzianum* showed the capability to tolerate Ni (Rosatto et al., 2019). Moreover, the Pikovskaya agar P solubilization test (Firew et al., 2016) and the Chrome Azurol siderophore production test (Milagres et al., 1999) highlighted PGP features (production of halos in the culture media) of the abovementioned species (Figure 1). Among microfungi, yeasts also can be PGP agents, but few works to date investigated their role. Fu et al. (2016) tested their yeast isolates for indole-3-acetic acid-, ammonia-, and polyamine-producing abilities, calcium phosphate and zinc oxide solubilizing ability, catalase activity, siderophore activity, and 1-aminocyclopropane-1-carboxylate deaminase, confirming the essential role that yeasts can play in the rhizosphere of plants.

## Interactions Among Microfungi and Bacteria in The Rhizosphere of Metallophytes

As shown in Table 1, few studies were carried out on the application of microfungi and bacteria in assisted phytoremediation (Jambon et al., 2018; Tiodar et al., 2021). Some researchers investigated the whole rhizospheric and endophytic microbiota, their roles in metal tolerance and transformation (Thijs et al., 2017), the efficiency of PGPB and arbuscular mycorrhizal in phytoremediation and the advantage of the synergistic application of fungi and bacteria (Kazemalilou et al., 2020). However, the fungi-bacteria interactions are complex to investigate (mainly by co-growth tests, culturable methods, or molecular methods such as metagenomic) and not completely understood (Deveau et al., 2018; Rosatto et al., 2021a). This latter can range from negative if, for example, they compete for nutrients; neutral if they do not interact; positive in case of synergistic relationships, for example, when microorganisms increase the nutrient bioavailability through nitrogen fixation and mobilization of key nutrients to the plants (Martin et al., 2017; Nazir et al., 2017; Deveau et al., 2018). For this reason, there is still much to investigate about the interaction between non-mycorrhizal rhizospheric microfungi and bacteria (Rashid et al., 2016). The study of interactions between these microorganisms dates back to the last 20 years, and its possible application to phytoremediation is very recent (Rashid et al., 2016). The synergistic interactions of various beneficial rhizosphere microbes could represent a potential suitable biotechnological tool for a successful phytoremediation process (Guarino and Sciarrillo, 2017). Often the employment of a single methodology of bioremediation is not enough for the total removal of contaminants in the medium and long term. Thanks to the research of new sustainable techniques of remediation, many studies on the selection of metallophytes and hyperaccumulator plants (Afif et al., 2021; Gieroń et al., 2021) have recently led to the investigation of the microbiota of the rhizosphere of these plants, trying to understand the mechanisms of metals tolerance, translocation, uptake, and the possible role played by associated roots microorganisms. In general, rhizosphere microbes of metallophytes and hyperaccumulator plants have been reported for the potential to increase in root surface area for the absorption and recycling of plant nutrients, improve plant health, and control plant pathogens (Figueiredo et al., 2010; Guarino and Sciarrillo, 2017). Guarino and Sciarrillo (2017) applied an integrated approach of bioremediation involving plants, autochthonous endo-ectomycorrhizal fungi, and bacteria. They explained that the inoculated plants showed an increase of biomass and toxic elements accumulation in the root system. To date, it is still questioned whether phytoremediation is attributed to the whole microbiome rather than a single taxon. Deng et al. (2018) tried to understand the role of the whole microbial community of *Elsholtzia splendens* Nakai ex F.Maek. var. *splendens* (Sub *E. haichowensis* Y.Z.Sun), a well-known indicator of Cu, under different environmental factors (EFs). They showed how these EFs can influence or not the microbial populations, giving a hand for understanding the construction of microbial communities in the rhizosphere. De Maria et al. (2011) studied the effect of phytoaugmentation with the rhizobacteria *Agromyces* sp., *Streptomyces* sp. and the co-inoculation of each of them with the fungus *Hyaloscypha finlandica* on biomass production and the bioaccumulation of selected trace elements (zinc [Zn], cadmium [Cd], and iron [Fe]) and macronutrients (calcium [Ca], potassium [K], phosphorus [P], and magnesium [Mg]) in *Salix caprea* L. grown on moderately polluted soil. They showed that the bacterial strain belonging to the *Streptomyces* genus was most efficient to increase the accumulation of Zn and Cd in leaves and shoots of *S. caprea*, but also that the combination of *H. finlandica* plus the bacterial strain of *Agromyces* genus resulted in an enhanced accumulation of Cd in shoots. *Streptomyces* is a well-known PGPB strain and the capability to favor the accumulation of the metal by *S. caprea* in the study (De Maria et al., 2011) confirmed it. However, recent investigations on the possible synergistic relations of this bacterium with rhizospheric fungi revealed a small possibility to interact with them. *Streptomyces* resulted to prefer to be indifferent to fungi or only developing slight positive interactions (Rosatto et al., 2021a). This feature seems to confirm that some strains of *Streptomyces* show a broad-spectrum antimicrobial activity (Worsley et al., 2020). Indeed, genus *Streptomyces* is well-known for its ability to synthesize a wide range of bioactive metabolites against bacteria, fungi, plants, insects, nematodes, and viruses (Hasani et al., 2014; Van der Meij et al., 2017; Hutchings et al., 2019).

**Table 1:** List of the main fungal-bacterial consortia employed for the attenuation of PTM stress and the improvement of phytoremediation efficiency in plants.

**Consortia**	**Plants**	**Matrices**	**References**
***Penicillium ochrochloron*** Biourge	*Alyssoides utriculata* (L.) Medik.	Soil naturally contaminated by Ni	Rosatto et al., 2021a
*Pseudomonas fluorescens* Migula 1895			
***Pisolithus arhizus*** (Scop.) Rauschert	*Acacia saligna* (Labill.) H.KL. Wendl.	Industrial site contaminated by PTMs	Guarino and Sciarrillo, 2017
***Acaulospora colombiana*** (Spain and N.C. Schenck) Kaonongbua, J.B. Morton and Bever			
***Rhizophagus clarus*** (T.H. Nicolson and N.C. Schenck) C. Walker and A. Schüßler			
***Claroideoglomus etunicatum*** (W.N. Becker and Gerd.) C. Walker and A. Schüßler			
***Rhizophagus intraradices*** (N.C. Schenck and G.S. Sm.) C. Walker and A. Schüßler			
*Bacillus licheniformis* (Weigmann 1898) Chester 1901			
*Priestia megaterium* (de Bary 1884) Gupta et al. 2020			
*Paenibacillus polymyxa* (Prazmowski 1880) Ash et al. 1994			
*Bacillus subtilis* (Ehrenberg 1835) Cohn 1872			
*Bacillus thuringiensis* Berliner 1915			
*Paenibacillus azotofixans* (Seldin et al. 1984) Ash et al. 1994			
***Pisolithus arhizus***	*Eucalyptus camaldulensis* Dehnh.	Industrial site contaminated by PTMs	Guarino and Sciarrillo, 2017
***Acaulospora colombiana***			
***Rhizophagus clarus***			
***Claroideoglomus etunicatum***			
***Rhizophagus intraradices***			
*Bacillus licheniformis*			
*Priestia megaterium*			
*Paenibacillus polymyxa*			
*Bacillus subtilis*			
*Bacillus thuringiensis*			
*Paenibacillus azotofixans*			
***Hyaloscypha finlandica*** (C.J.K. Wang and H.E. Wilcox) Vohník, Fehrer, and Réblová	*Salix caprea* L.	Experimental soil contaminated by PTMs	De Maria et al., 2011
*Agromyces* sp.			
*Streptomyces* sp.			
***Paecilomyces formosus*** Sakag., May. Inoue and Tada ex Houbraken and Samson	*Glycine max* L.	Experimental soil contaminated by Al and Zn	Bilal et al., 2018
*Sphingomonas* sp.			
***Glomus*** sp.	*Cenchrus americanus* (L.) Morrone	Soil contaminated by Fe	Mishra et al., 2016
***Acaulospora*** sp.			
***Scutellospora*** sp.			
*Streptomyces* sp.			
*Azotobacter* sp.			
*Pseudomonas* sp.			
*Paenibacillus* sp.			
***Glomus*** sp.	*Sorghum bicolor* (L.) Moench	Soil contaminated by Fe	Mishra et al., 2016
***Acaulospora*** sp.			
***Scutellospora*** sp.			
*Streptomyces* sp.			
*Azotobacter* sp.			
*Pseudomonas* sp.			
*Paenibacillus* sp.			
***Acaulospora mellea*** Spain and N.C. Schenck	*Centrosema coriaceum* Benth.	Iron ore deposit	Matias et al., 2009
***Gigaspora margarita*** W.N. Becker and I.R. Hall			
***Dentiscutata heterogama*** (T.H. Nicolson and Gerd.) Sieverd., F.A. Souza and Oehl			
***Glomus*** sp.			
***Fusarium oxysporum*** Schltdl.			
***Aspergillus fischeri*** Wehmer			
***Rhizobium*** sp.			
***Acaulospora mellea***	*Pleroma heteromallum* (D.Don) D.Don	Iron ore deposit	Matias et al., 2009
***Gigaspora margarita***			
***Dentiscutata heterogama***			
***Glomus*** sp.			
***Fusarium oxysporum***			
***Aspergillus fischeri***			
***Rhizobium*** sp.			

*Fungal taxa are in bold, and bacterial taxa are underlined. The taxonomy of fungi, bacteria, and plants is according to Index Fungorum (http://www.indexfungorum.org), APIWEB (https://www.biomerieux.it), and Plants of the World Online (http://www.plantsoftheworldonline.org)*.

Another study by Rosatto et al. (2021a,b) was conducted on the root development with respect to metals and possible interactions of bacteria and microfungal strains isolated from the rhizosphere of the facultative hyperaccumulator *Alyssoides utriculata*, showing how only a few species built positive relations and co-grew with it. The best performing strains in terms of Ni tolerance and PGP traits were subjected to *in vitro* tests to establish mutual interactions and identify a clear synergy between the bacterial and the fungal component (Rosatto et al., 2021a). Thanks to their tests, they selected a *P. fluorescens* strain and a *Penicillium ochrochloron* strain as the most performant. Their synergistic behavior could suggest the potential use of *in vivo* microorganism consortia to mitigate metal stress and promote metal uptake for bioremediation purposes.

## Conclusion

The development of integrated approaches of bioremediation, which involve different synergistic organisms together, is the new frontier of bioremediation techniques. Hence, the rhizobiota of metallophytes seems to play a central role as a basin of PGP bacteria and microfungi employable in phytoremediation processes. However, to date, many studies should be carried out to deepen and understand the interactions among roots, bacteria, and microfungi in the tolerance and uptake of PTMs. It has been shown that microorganisms associated with the roots of metallophytes can individually increase the plant biomass of the roots themselves (Wu et al., 2020b,c), while there are few studies that demonstrate how both bacteria and microfungi can further increase root biomass together.

## Author Contributions

GC, SR, MM, ER, and MZ contributed to conception of the paper and organized the data. GC and SR wrote the first draft of the manuscript. GC, SD, SR, and ER wrote sections of the manuscript. All authors contributed to manuscript revision, read, and approved the submitted version.

## Conflict of Interest

The authors declare that the research was conducted in the absence of any commercial or financial relationships that could be construed as a potential conflict of interest.

## Publisher's Note

All claims expressed in this article are solely those of the authors and do not necessarily represent those of their affiliated organizations, or those of the publisher, the editors and the reviewers. Any product that may be evaluated in this article, or claim that may be made by its manufacturer, is not guaranteed or endorsed by the publisher.
